# S-Adenosyl-l-Methionine Overcomes uL3-Mediated Drug Resistance in p53 Deleted Colon Cancer Cells

**DOI:** 10.3390/ijms22010103

**Published:** 2020-12-24

**Authors:** Laura Mosca, Martina Pagano, Annalisa Pecoraro, Luigi Borzacchiello, Luigi Mele, Giovanna Cacciapuoti, Marina Porcelli, Giulia Russo, Annapina Russo

**Affiliations:** 1 Department of Precision Medicine, University of Campania “Luigi Vanvitelli”, Via Luigi De Crecchio, 80138 Naples, Italy; laura.mosca@unicampania.it (L.M.); luigi.borzacchiello@unicampania.it (L.B.); giovanna.cacciapuoti@unicampania.it (G.C.); 2 Department of Pharmacy, University of Naples “Federico II”, Via Domenico Montesano 49, 80131 Naples, Italy; martina.pagano@unicampania.it (M.P.); annalisa.pecoraro@unina.it (A.P.); 3 Department of Experimental Medicine, University of Campania “Luigi Vanvitelli”, Via Luciano Armanni, 5, 80138 Naples, Italy; luigi.mele@unicampania.it

**Keywords:** uL3, drug resistance, AdoMet, autophagy, apoptosis, colon cancer

## Abstract

Purpose: In order to study novel therapeutic approaches taking advantage of natural compounds showing anticancer and anti-proliferative effects, we focused our interest on S-adenosyl-l-methionine, a naturally occurring sulfur-containing nucleoside synthesized from adenosine triphosphate and methionine by methionine adenosyltransferase, and its potential in overcoming drug resistance in colon cancer cells devoid of p53. Results: In the present study, we demonstrated that S-adenosyl-l-methionine overcomes uL3-mediated drug resistance in p53 deleted colon cancer cells. In particular, we demonstrated that S-adenosyl-l-methionine causes cell cycle arrest at the S phase; inhibits autophagy; augments reactive oxygen species; and induces apoptosis in these cancer cells. Conclusions: Results reported in this paper led us to propose S-adenosyl-l-methionine as a potential promising agent for cancer therapy by examining p53 and uL3 profiles in tumors to yield a better clinical outcomes.

## 1. Introduction

Colon cancer is one of the most common causes of cancer-related death worldwide and its incidence is steadily rising [1]. Surgery, chemotherapy, and radiotherapy, either individually or in combination, are recognized as key treatments for all stages of colon cancer and are crucial in determining patient survival and quality of life. Although, chemotherapy is very effective after surgery, the clinical outcome of this therapeutic approach can be unsatisfactory, especially in patients with advanced disease or in the presence of metastasis [2]. In cancer therapy, the development of intrinsic or acquired drug resistance and/or multidrug resistance (MDR) is the leading cause of colon tumor recurrence [3]. Thus, recent studies have yielded important information on how to overcome the resistance improving cancer chemotherapy and this field remains one of the major challenges.

Over the past decades, several studies have reported a role for ribosomal proteins (RPs) in multidrug resistance. To date, the upregulation of uL4 and uL18 was associated with doxorubicin resistance in human colon cancer cells [4]; higher intracellular levels of eS28 was associated with acquired cisplatin resistance in human head and neck carcinoma cell lines [5]. Other studies have reported the high expression of eL8 and eL6 in adriamycin-resistant gastric cancer cells [6]. Shi et al. have demonstrated the involvement of uS15 and uL14 in the development of resistance to vincristine, adriamycin, and 5-fluorouracil (5-FU) in a multidrug-resistant gastric cancer cell line SGC790 [7].

RPs also have a role in the regulation of molecular mechanisms underlying the development of drug resistance in cancer cells that contain mutant p53 or no p53 at all [8]. Among these, we have recently identified a specific pathway depending on uL3 status in colon and lung cancer devoid of p53 [9,10,11].

uL3 is a member of a subset of RPs that, as the ribosome-free form, are implicated in various important events in cell life [12]. In particular, uL3 regulates its own expression through the generation of mRNA isoforms that are the target of nonsense-mediated mRNA decay [13,14].

We demonstrated that the expression of uL3 is downregulated in colon tumor tissues [15] and that uL3 overexpression stimulated apoptotic cell death by inducing late apoptosis [16]. In particular, we showed that uL3ΔHCT 116^p53−/−^ colon cancer cells in which uL3 levels were strongly lower than those in the parental cell line [17] resulted in being resistant to different chemotherapeutic agents including 5-FU, oxaliplatin, and actinomycin D [17,18,19,20]. The reduction of uL3 levels in these cells is associated with alteration in cell motility and the epithelial mesenchymal transition (EMT) phenotype, a migratory cellular program strictly interconnected with tumor development and metastasis [10].

How to overcome drug resistance is a key issue for cancer treatment. Emerging evidence indicates that natural compounds are a promising source of new anticancer drugs.

S-Adenosyl-l-methionine (AdoMet) is a major cellular methyl donor and exerts a primary role in several biochemical reactions, making it the second most used enzyme cofactor after ATP [21,22,23]. AdoMet plays a critical role in the synthesis of polyamines, provides cysteine for the production of glutathione, and is involved in 5′-deoxyadenosyl-5′-radical-mediated biochemical transformations and in the biosynthesis of different metabolites [21,24,25,26,27].

Aside from its well-known functions, AdoMet exerts antitumor action through different biological pathways depending on cancer cell-type [26,27,28,29,30,31,32,33]. Hence, many in vitro and in vivo studies showed the involvement of the sulfonium compound in the regulation of cell growth, induction of apoptosis, autophagy, unfolding protein response, and inhibition of invasion and metastatic spread in different kinds of human cancer [27,28,29,30].

Here, we demonstrated that AdoMet overcame uL3-mediated drug resistance in uL3ΔHCT 116^p53−/−^ colon cancer cells. We reported that AdoMet-induced antiproliferative effects were accompanied by cell cycle arrest in the S-phase, inhibition of autophagy, increase of ROS, and induction of apoptosis. AdoMet consistently caused a strong decrease of cyclin D1 levels and a marked increase of pro-apoptotic caspase 3/8/9 activation and PARP-1 cleavage. Altogether, this study provides a new exploratory perspective for overcoming the drug resistance of colon cancer cells showing p53 and uL3 lower levels by using the biomolecule AdoMet.

## 2. Results

### 2.1. AdoMet Inhibits Proliferation in HCT 116^p53−/−^ and uL3ΔHCT 116^p53−/−^ Colon Cancer Cells

**Figure 1:**
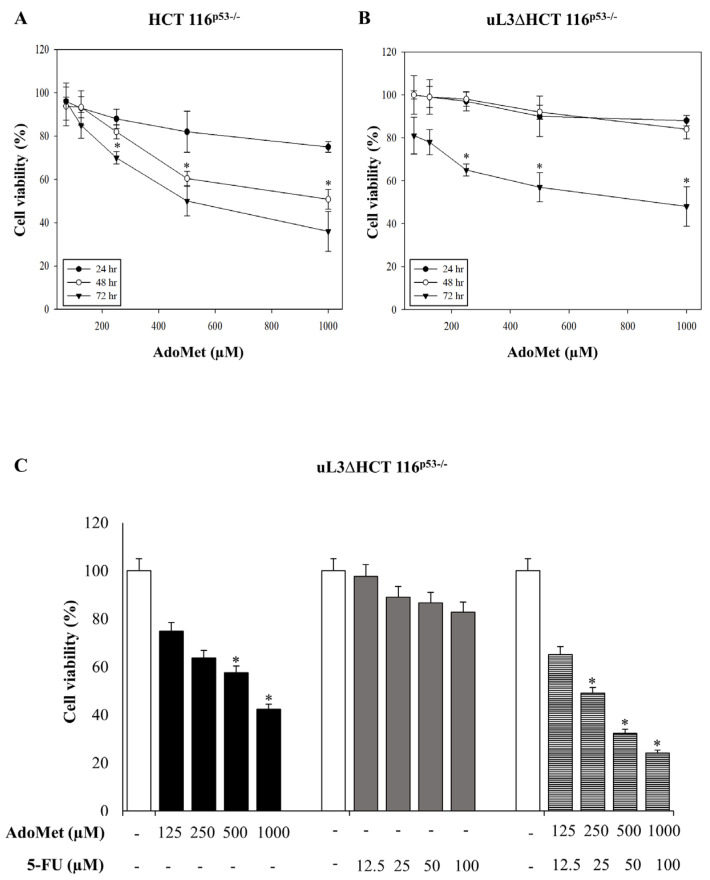
Cytotoxic effect of S-Adenosyl-l-methionine (AdoMet) on HCT 116^p53−/−^ and uL3ΔHCT 116^p53−/−^ colon cancer cells. HCT 116^p53−/−^ (**A**) and uL3ΔHCT 116^p53−/−^ cells (**B**) were treated with increasing amounts of AdoMet (72–1000 µM) from 24 to 72 h, then cell viability was measured by the MTT assay. Results are presented as a percentage of the control cells. * *p* < 0.05 versus untreated cells (**C**) uL3ΔHCT 116^p53−/−^ were treated with AdoMet (from 125 to 1000 µM), and 5-FU (from 12.5 to 100 µM), alone or in combination, for 72 h. Cell viability was assayed using the MTT assay. Data represent the average of three independent experiments; error bars represent the standard deviation. * *p* < 0.05 versus untreated cells.

With the aim to investigate the role of AdoMet in uL3-mediated drug resistance, we first evaluated its effects on cell proliferation in our model of colon cancer cells deleted of p53 [17]. To this aim, sensitive HCT116^p53−/−^ cells and drug-resistant uL3ΔHCT116^p53−/−^ cells were incubated with different concentrations (from 72 to 1000 µM) of AdoMet. The 24, 48, and 72 h later cell viability was assessed by the 3-(4,5-dimethylthiazol-2-yl)-2,5-diphenyltetrazolium bromide (MTT) assay. As shown in Figure 1A, HCT 116^p53−/−^ cells showed sensitivity to AdoMet and cell viability was significantly decreased in a dose- and time-dependent manner. These data demonstrated that AdoMet exerted cytotoxic effects in HCT 116^p53−/−^ colon cancer cells. Of interest, AdoMet also showed cytotoxic activity in resistant uL3ΔHCT 116^p53−/−^ cells (Figure 1B), although with lower efficacy as demonstrated by an IC_50_ value of 750 µM compared to an IC_50_ value of 500 µM in HCT 116^p53−/−^ cells at 72 h. These data suggest that AdoMet could restore the sensitivity of resistant uL3ΔHCT 116^p53−/−^ cells. We have previously demonstrated that in these cells, the silencing of uL3 completely abolished the cytotoxicity of 5-FU treatment [17]. To demonstrate the crucial role of AdoMet in overcoming uL3-mediated drug resistance, we investigated the role of AdoMet alone or in combination with 5-FU on cell viability. To this purpose, uL3ΔHCT 116^p53−/−^ cells were treated with AdoMet (from 125 to 1000 µM), 5-FU (from 12.5 to 100 µM), or with a combination of 5-FU and AdoMet. For the 72 h later, cell viability was estimated by the 3-(4, 5-dimethylthiazol-2-yl)- 2, 5-diphenyltetrazolium bromide (MTT) assay. As expected, the percentage of cell viability decreased after AdoMet treatment while the treatment with 5-FU had no effect on cell viability. Of note, in cells treated with 5-FU plus AdoMet, we found that the cell viability was strongly reduced compared to that of cells exposed to AdoMet alone (Figure 1C). Altogether, these data show that AdoMet re-sensitizes drug resistant colon cancer cells to 5-FU treatment.

### 2.2. AdoMet Causes Cell Cycle Arrest in S Phase in uL3ΔHCT 116^p53−/−^

**Figure 2:**
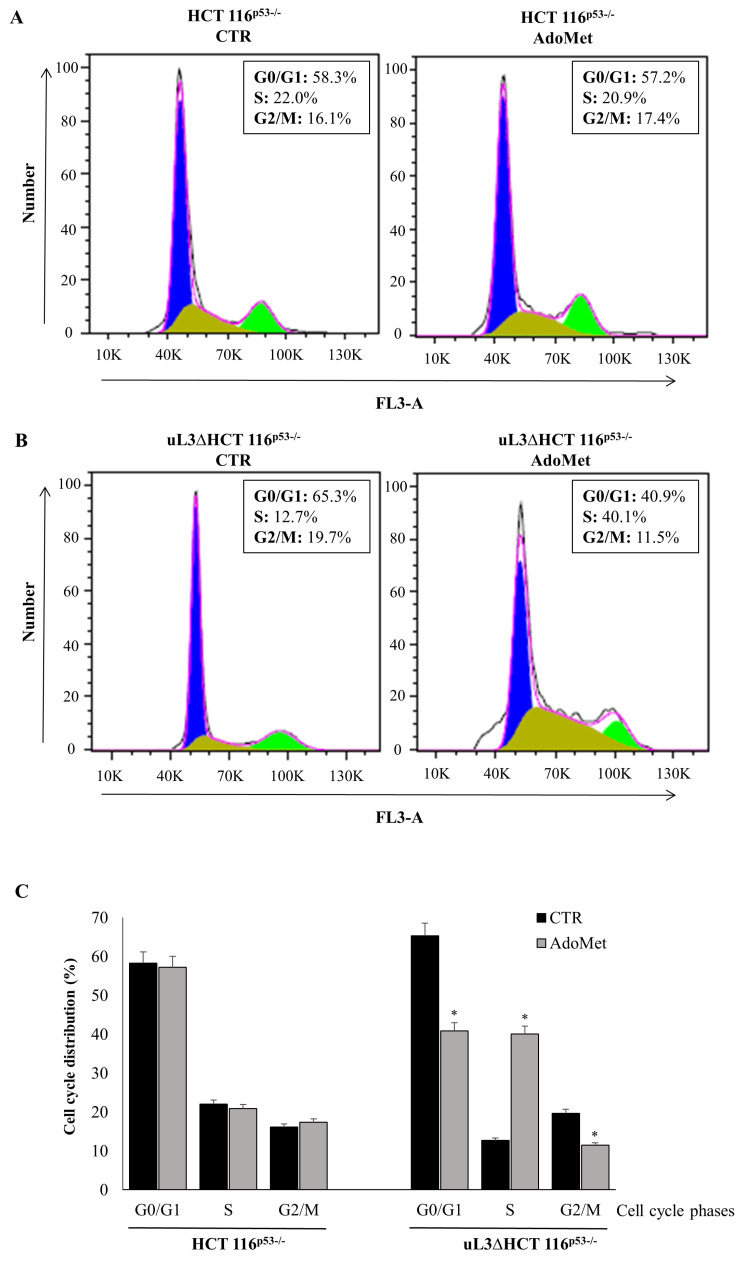
Effect of AdoMet on cell cycle in HCT 116^p53−/−^ and uL3ΔHCT 116^p53−/−^ colon cancer cells. Representative FACS histograms of propidium iodide (PI)-stained of HCT 116^p53−/−^ (**A**) and uL3ΔHCT 116^p53−/−^ (**B**) cells treated or not (CTR) with 500 µM AdoMet for 72 h. (**C**) The bar diagram shows the percentage of cells in each phase of the cell cycle. Each bar represents the mean of triplicate experiments; error bars represent the standard deviation. For each sample, at least 2 × 10^4^ events were analyzed. * *p* < 0.05 versus untreated cells.

**Figure 3:**
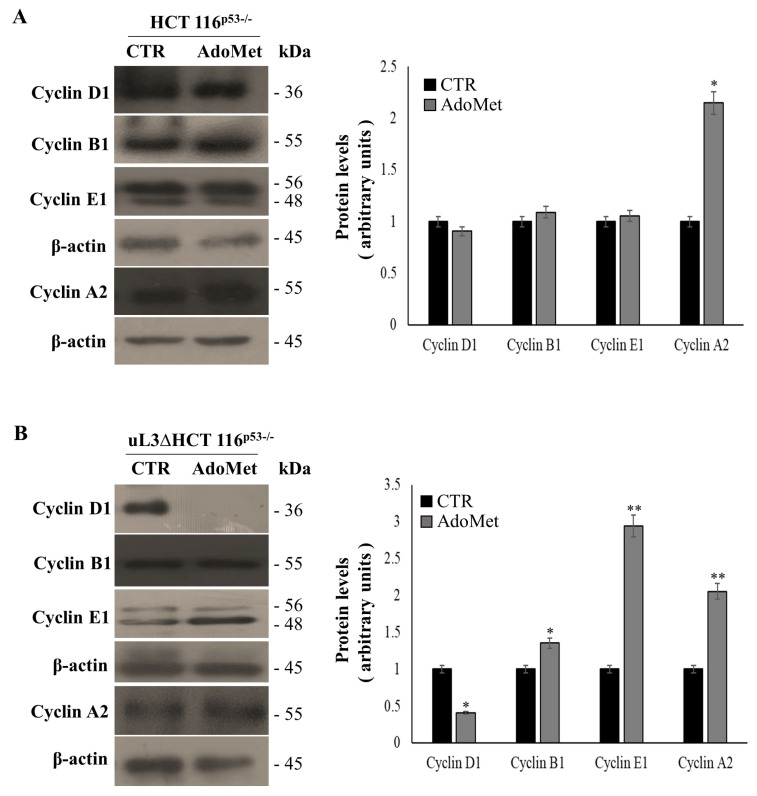
Effect of AdoMet on cell cycle regulatory proteins in HCT 116^p53−/−^ and uL3ΔHCT 116^p53−/−^ colon cancer cells. The level of cell cycle-regulatory proteins in HCT 116^p53−/−^ (**A**) and uL3ΔHCT 116^p53−/−^ cells (**B**) was measured by western blot analysis. The housekeeping protein β-actin was used as a loading control. The relative densitometric analysis is reported in the corresponding graphs, expressed as arbitrary units. Bars represent the mean of triplicate experiments; error bars represent the standard deviation. * *p* < 0.05, ** *p* < 0.01 vs. untreated cells set at 1. The images are representative of three immunoblotting analyses obtained from three independent experiments. Full-length blots are shown in Figure S1.

In order to investigate the mechanism by which AdoMet exerts its cytotoxic activity in p53 deleted colon cancer cells, alterations in the cell cycle distribution were analyzed. To this aim, HCT 116^p53−/−^ and uL3ΔHCT 116^p53−/−^ cells were treated with 500 µM of AdoMet. Seventy-two hours later, cell cycle distribution was monitored by flow cytometry. Analysis of cell cycle profile of treated HCT 116^p53−/−^ cells showed that AdoMet had no effect (Figure 2A,C). To confirm this result, the expression levels of key regulators of the cell cycle phases were assessed. Consistently, the expression profile of cyclin B, D, and E resulted in being unaltered upon AdoMet treatment, while the expression levels of cyclin A were increased (Figure 3A). Interestingly, the observed upregulation of cyclin A could be involved in mechanisms of cell death as already reported [34]. In the absence of uL3, the exposure of cells to AdoMet caused an increased percentage of cell population in the S phase (from about 13% to about 40%) with a relative decreased cell population in the G2/M phase (from about 20% to about 11%) and G1 phase (from about 65% to about 41%) (Figure 2B,C). Analysis of cyclin level in these cells showed that AdoMet treatment caused an upregulation of the G1 phase-related protein as cyclin E and G2/M phase-related cyclin B. Furthermore, as observed in HCT 116^p53−/−^, the levels of cyclin A increased following AdoMet treatment (Figure 3B). More importantly, the treatment of uL3ΔHCT 116^p53−/−^ cells with AdoMet was associated with a marked downregulation of cyclin D that, as we have previously demonstrated [10], is upregulated in these cells as aa consequence of uL3 silencing.

All these data indicate that the inhibitory effects of AdoMet on uL3ΔHCT 116^p53−/−^ cell proliferation were associated with the alteration in cell cycle progression, downregulation of cyclin D1, and upregulation of Cyclin A.

### 2.3. AdoMet Induces Apoptosis in HCT 116^p53−/−^ and uL3ΔHCT 116^p53−/−^ Colon Cancer Cells

**Figure 4:**
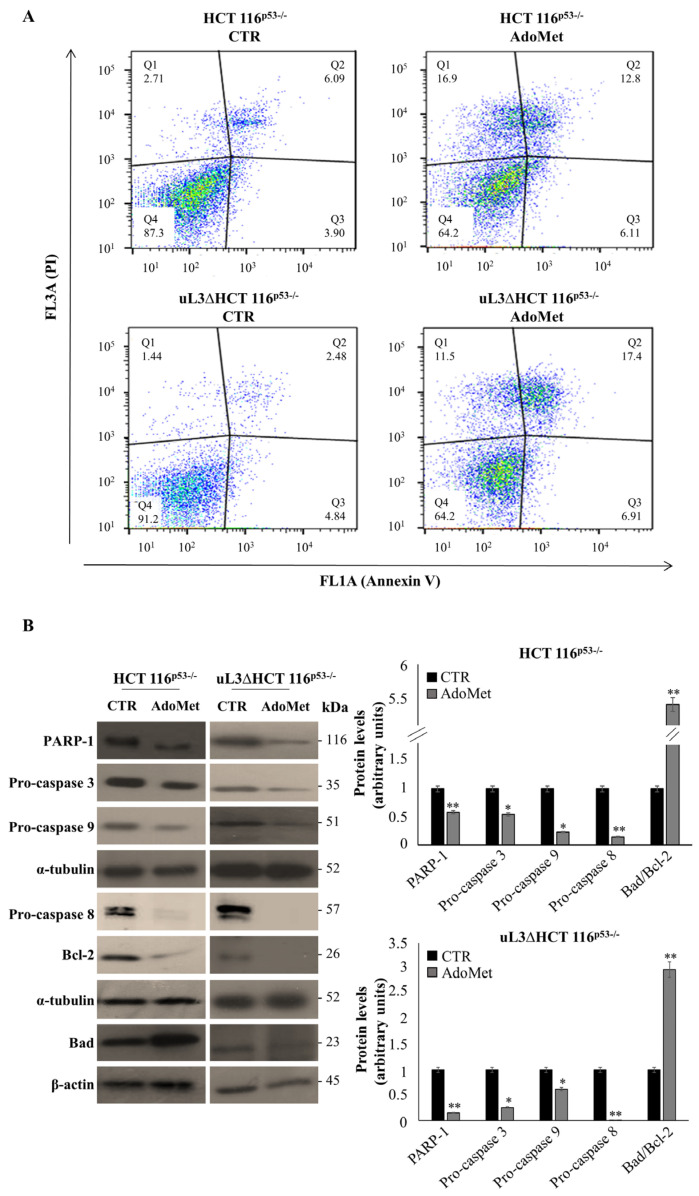
Effect of AdoMet on apoptosis in HCT 116^p53−/−^ and uL3ΔHCT 116^p53−/−^ colon cancer cells. (**A**) Representative dot plots of Annexin V-FITC and PI-stained HCT 116^p53−/−^ and uL3ΔHCT 116^p53−/−^ cells incubated with 500 μM AdoMet for 72 h. The experiments were repeated three times. (**B**) Immunoblots of HCT 116^p53−/−^ and uL3ΔHCT 116^p53−/−^ for PARP-1, pro-caspase 3, pro-caspase 8, pro-caspase 9, Bad, and Bcl-2; the housekeeping proteins α-tubulin and β-actin were used as loading control. Graphs show the relative densitometric analyses, expressed as arbitrary units. Bars represent the mean of triplicate experiments; error bars represent the standard deviation. * *p* < 0.05, ** *p* < 0.01 vs. untreated cells set at 1. The images are representative of three immunoblotting analyses obtained from three independent experiments. Full-length blots are shown in Figure S2.

To further investigate the underlying mechanism that contributes to AdoMet-mediated growth inhibition in our model of colon cancer cells, we determined whether AdoMet decreased cell survival through the induction of apoptosis. To this aim, Annexin V analysis was performed. HCT 116^p53−/−^ cells and uL3ΔHCT 116^p53−/−^ cells were treated with 500 µM AdoMet and 72 h after were assessed with Annexin V-FITC. As shown in Figure 4A, the percentage of Annexin-V positive cells increased after AdoMet treatment compared to untreated cells in both cell lines. Specifically, we found that in HCT 116^p53−/−^ cells, AdoMet significantly increased the percentage of late apoptotic cells (Annexin V+ and PI+) from 6% in the untreated cells to 13% in the treated cells (Figure 4A). Upon exposure to AdoMet in the uL3ΔHCT 116^p53−/−^ cell line, apoptotic cells proportion raised from 3 to 17% (Figure 4A). Since it is well-recognized that caspase family and PARP-1 play pivotal roles in the initiation and execution of apoptosis, the expression of these apoptosis-related proteins was assessed by western blot analysis. As expected, Figure 4B showed the downregulation of PARP-1, pro-caspase 3, 9, 8, and an increase in the ratio between pro-apoptotic Bad and anti-apoptotic Bcl-2 in both cell lines [35].

### 2.4. AdoMet Increases ROS Production in HCT 116^p53−/−^ and uL3ΔHCT 116^p53−/−^ Colon Cancer Cells

Since many anticancer drugs and natural compounds have been highlighted to exert a pro-apoptotic effect in neoplastic cells due to their pro-oxidant activity, we were interested in evaluating the levels of reactive oxygen species (ROS) in colon cancer cells in the presence and absence of uL3. Therefore, HCT 116^p53−/−^ cells and uL3ΔHCT 116^p53−/−^ cells were treated with 500 μM AdoMet for 72 h and the intracellular ROS levels were quantitatively evaluated by the 2′,7′-dichlorofluorescein diacetate (DCF-DA) flow cytometric assay.

**Figure 5:**
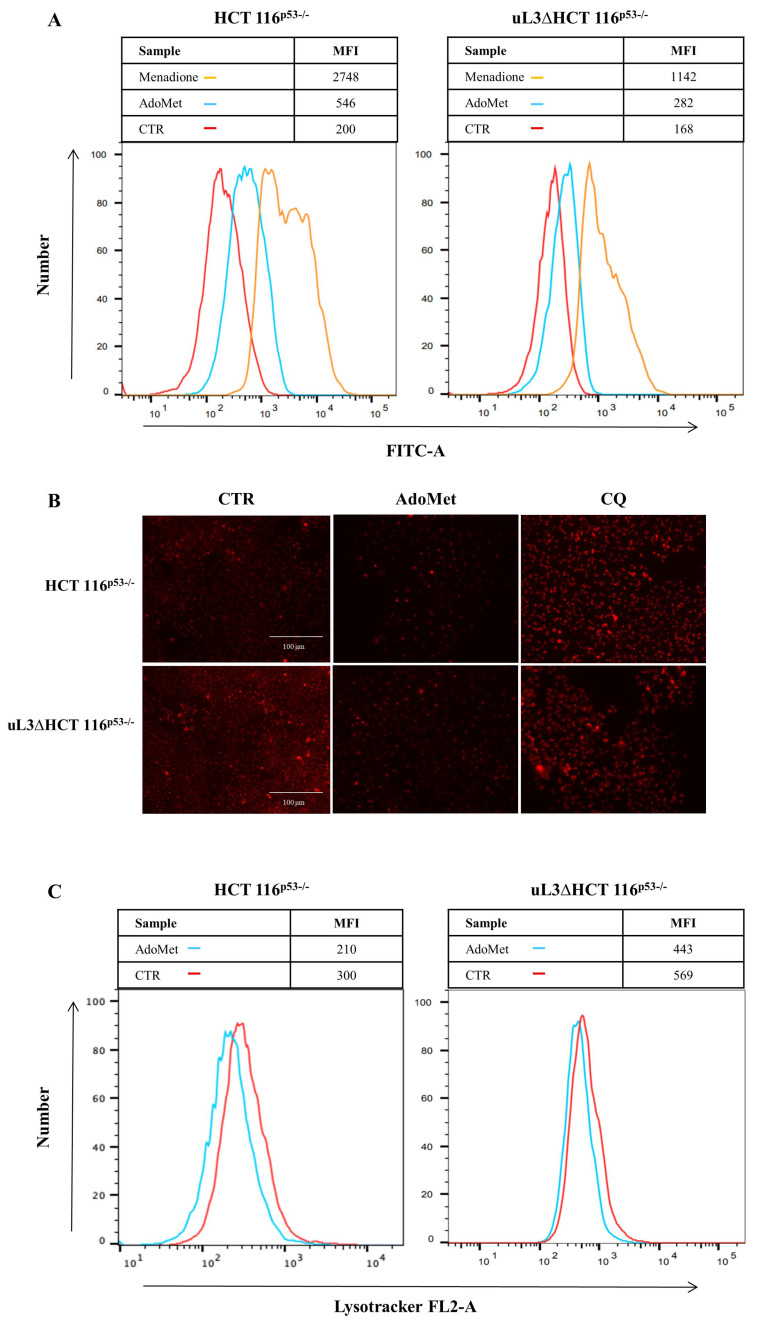
Effect of AdoMet on ROS accumulation and on autophagic flux in HCT 116^p53−/−^ and uL3ΔHCT 116^p53−/−^ colon cancer cells. (**A**) HCT 116^p53−/−^ and uL3ΔHCT 116^p53−/−^ cells were treated or not (CTR) with 500 μM AdoMet for 72 h and than subjected to flow cytometry to measure ROS levels. FACS analysis was performed using 2′,7′-dichlorofluorescein diacetate (DCF-DA) as a substrate. For the quantitative evaluation of ROS, FlowJo software was used to calculate median fluorescence intensities (MFI) by the formula (MFI-treated/MFI-control). Analysis was carried out by triplicate determination on three separate experiments. (**B**) Representative images of vital dye LysoTracker (LTR) staining of HCT 116^p53−/−^ and uL3ΔHCT 116^p53−/−^ cells treated or not (CTR) with 500 μM of AdoMet for 48 h analyzed by fluorescence microscopy. Chloroquine (CQ) was used as a positive control. (**C**) Flow cytometry analysis of HCT 116^p53−/−^ and uL3ΔHCT 116^p53−/−^ labeled with LTR. Median fluorescence values are shown in the graphs. At least 2 × 10^4^ events were acquired in log mode. For the quantitative evaluation of LTR, FlowJo software was used to calculate median fluorescence intensities (MFI) by the formula (MFI-treated/MFI-control). The experiments were repeated three times.

Results in Figure 5A showed an increase in ROS production after AdoMet treatment in both cell lines, as evidenced by the reported median intensity fluorescence (MFI) value. Specifically, an increase of 2- and 3-fold in ROS levels was observed in uL3∆HCT 116^p53−/−^ and HCT 116^p53−/−^ cells, respectively, compared to the control cells. Overall, these results indicate that the activation of ROS signaling could represent one of the mechanisms underlying AdoMet-induced apoptosis in these cell lines.

### 2.5. AdoMet Inhibits Autophagy in HCT 116^p53−/−^ and uL3ΔHCT 116^p53−/−^ Colon Cancer Cells

Autophagy plays a role as a pro-survival mechanism for drug resistance [36]. We have recently demonstrated that the chemoresistance observed in uL3∆HCT 116^p53−/−^ cells might depend on the higher autophagic flux displayed by this cell population [11]. To investigate if the responsivity of uL3∆HCT 116^p53−/−^ cells to AdoMet exposure may be correlated with autophagy modulation, we were interested in evaluating the autophagy occurrence in treated cells. To this aim, HCT 116^p53−/−^ and uL3∆HCT 116^p53−/−^ cells were treated with 500 μM AdoMet and 48 h later were stained with vital dye LysoTracker (LTR), a fluorescent dye for labeling and tracking cellular acidic compartments, lysosomes, and autophagolysosomes. As shown in Figure 5B, fluorescence microscopy analysis revealed a decreased formation of red dotted acidic vacuoles in comparison with untreated cells (CTR), providing evidence of autophagic flux inhibition caused by AdoMet treatment in both cell lines. To further characterize the effect of AdoMet, flow cytometry analysis was performed in the same cells. Results in Figure 5C showed a decrease in acidic vesicles after treatment with AdoMet as assessed by measuring median fluorescence in both cell lines.

One important step in autophagosome formation involves the cleavage of LC3B-I to LC3B-II by the coordinated activity of several proteins including Atg7 and subsequent association of LC3B-II with autophagosomal membranes. Thus, p62 binds directly to ubiquitinated proteins and gets degraded during active autophagy [37].

**Figure 6:**
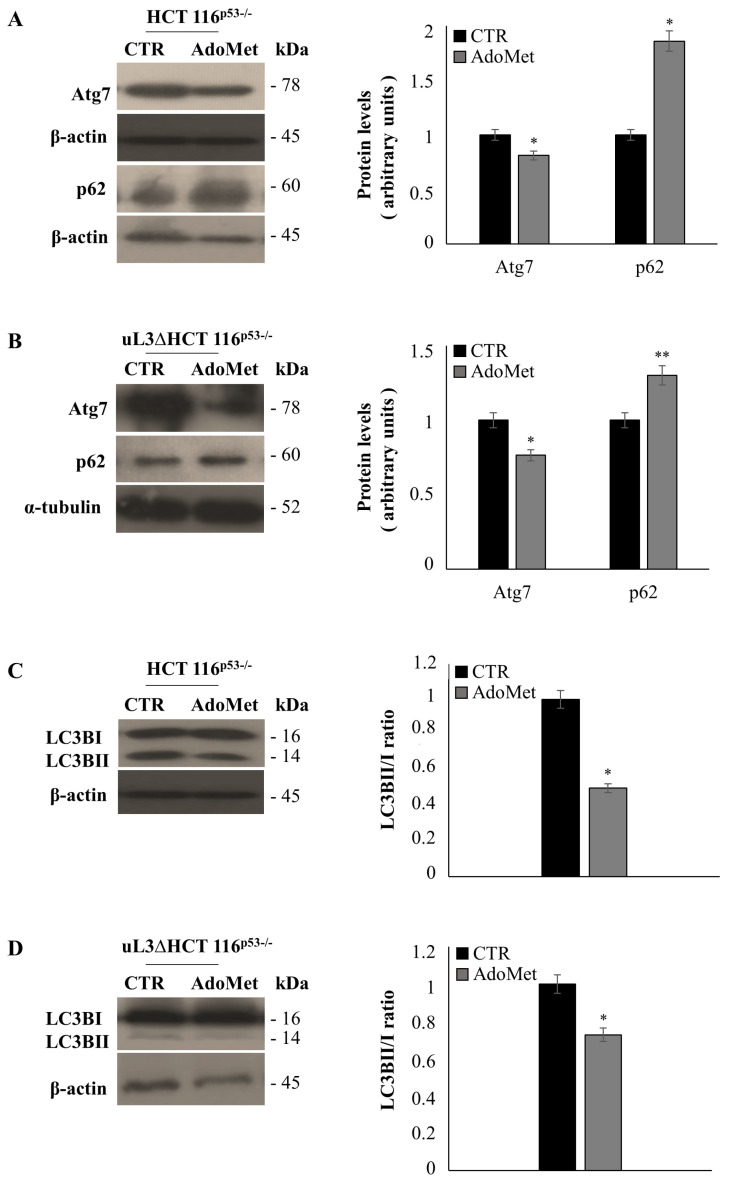
Effect of AdoMet on the autophagy related markers in HCT 116^p53−/−^ and uL3ΔHCT 116^p53−/−^ colon cancer cells. Representative immunoblotting of ATG7, p62, and LC3BI/II in HCT 116^p53−/−^ cells (**A**,**C**) and uL3ΔHCT 116^p53−/−^ cells (**B**,**D**) treated or not with 500 μM AdoMet for 48 h. The housekeeping protein β-actin was used as a loading control; graphs show the relative densitometric analyses, expressed as arbitrary units. Quantification of the LC3B-II/I ratio is shown in the graphs. Bars represent the mean of triplicate experiments; error bars represent the standard deviation. * *p* < 0.05, ** *p* < 0.01 vs. untreated cells set at 1. Full-length blots are shown in Figure S3.

To further demonstrate the involvement of autophagy in the AdoMet response of colon cancer cells, and in particular, the ability of AdoMet to overcome the drug resistance of uL3∆HCT 116^p53−/−^ cells, we evaluated the protein expression levels of Atg7, p62, and LC3B by western blot analysis in both cell lines. Figure 6A,B showed that AdoMet treatment caused a reduction of Atg7 and an increase in p62 levels in both cell lines, which was associated with a decrease in LC3BII/I ratio (Figure 6C,D).

These findings confirm the inhibitory effect of AdoMet on autophagic flux in both colon cancer cell lines.

## 3. Discussion

The failure of conventional anticancer therapy is mainly due to the development of drug resistance that represents a serious problem in the treatment of cancer. Although many mechanisms of tumor chemoresistance have been discovered and elucidated, it remains the leading cause of chemotherapy failure, especially in relapsed or metastatic tumors. Re-sensitizing cancer cells to the drugs by using novel effective strategies is a promising approach to overcome the chemoresistance and improve clinical treatment [38,39]. Recently, we evaluated uL3 clinical significance in colon cancer and demonstrated that uL3 expression in colon tumor tissues is downregulated; in particular, the decrease in uL3 mRNA levels associated with malignance progression and tumor grade and were inversely proportional to the ratio Bcl-2/Bax [15]. Loss of uL3 in p53-deleted colon cancer cells was associated with characteristic invasive phenotype with EMT and correlated with the development of resistance to different chemotherapeutic agents commonly used in clinic for the treatment of colon cancer as 5-FU [9,10,15].

Depletion of uL3 confers drug resistance through the alteration of various signaling pathways regulating cell cycle, cell migration, apoptosis, and autophagy [8,40]. Different approaches have been proposed to come up with effective drugs to overcome drug resistance. Combination of different drugs attacking multiple pathways simultaneously presents some limitations including deleterious effects from drug–drug interactions, overlapped toxicity, and costs of combination therapy [38]. Moreover, accumulating evidence suggests that simultaneous inhibition of multiple signaling pathways by natural compounds could represent a better therapeutic approach than that of individual inhibitors.

Over the past two decades, the in vitro and in vivo impacts of AdoMet against the development and the progression of many types of human cancer has been demonstrated through its involvement in different biological pathways [26,27,28,29,30,31,32,33]. In this regard, AdoMet has become particularly attractive, taking advantage of its pleiotropic effects on different survival pathways known to be important in colon carcinogenesis and could represent a promising strategy to overcome uL3-mediated drug resistance.

AdoMet has been shown to induce the apoptotic process and reduce the mechanism of inflammation and invasion in colon cancer cells [41,42,43,44,45]. In RKO and HT-29 colon cancer cells, AdoMet treatment reduced the expression of several anti-apoptotic genes such as the gene encoding FLICE-like inhibitory protein and caused the activation of caspase 8 with the consequent release of cytochrome from the mitochondria [41].

Luo et al. reported that in colon cancer cells, AdoMet inhibited the tumor cell growth by reversing the DNA hypomethylation on promoters of c-Myc and H-ras oncogenes, thus downregulating their expression [42]. Furthermore, AdoMet was able to prevent the inflammation-induced colon cancer by azoxymethane and dextran sulfate sodium in Balb/c mice through inhibition of β-catenin, IL-6 signaling, pro-inflammatory, pro-growth, and proliferation pathways [43].

In the highly invasive SW-620 colorectal cancer cell line, the sulfonium compound reduces the metastatic process by the hypermethylation of specific genes like the matrix metalloproteinase-2 and the membrane type 1 matrix metalloproteinase [44]. Recently, in colon SW480 and HCT116 cancer cells with constitutively active β-catenin signaling, AdoMet treatment inhibited β-catenin activity, preventing its access to the nuclear compartment. Moreover, in colon RKO cancer cells expressing wild-type Wnt/β-catenin, AdoMet enhanced GSK3β-mediated degradation of β-catenin by elevating the activity of protein phosphatase 2A [45]. In this regard, thanks to its high safety profile, AdoMet represents a hopeful strategy to overcome uL3-mediated drug resistance, without the common side effects of chemotherapy drugs [33].

In this study, we focused our study on examining the therapeutical potential of AdoMet to overcome drug resistance in the uL3∆HCT 116^p53−/−^ cell line, a model of colon cancer cells resistant to conventional anticancer drugs including 5-FU, based on the absence of p53 and low levels of uL3 [10]. Results of cytotoxicity experiments demonstrated that AdoMet caused cell death in uL3∆HCT 116^p53−/−^ cells in a dose- and time-dependent manner. Of note, the combined treatment of resistant cells with AdoMet plus 5-FU was able to rescue the cytotoxic effects of 5-FU. These data strongly demonstrate the key role of AdoMet in overcoming uL3 mediated drug resistance. To our knowledge, the present study demonstrates, for the first time, evidence that AdoMet treatment re-sensitizes drug resistant colon cancer cells. These data raise the possibility that AdoMet might be a potential therapeutic agent for resistant colon cancer cells showing a low expression profile of p53 and uL3. In light of these observations, we were interested to exploring the mechanism underlying AdoMet-induced cell death.

Cell cycle progression and its regulation are the key events of cell proliferation. In HCT 116^p53−/−^ cells, AdoMet treatment did not affect cell cycle profile, as confirmed by the analysis of the expression profile of cell cycle related proteins, indicating that the observed cytotoxic effects were not due to the deregulation of cell cycle checkpoints. Although no apparent modifications of the expression profile of most cyclins was evidenced, an increase of cyclin A level was observed upon AdoMet treatment. Recent findings highlighted cyclin A involvement in the triggering of apoptosis, suggesting that the observed activation of the apoptotic process by AdoMet in HCT 116^p53−/−^ cells could be due to the alteration of cyclin A expression [34].

Of interest, in uL3∆HCT 116^p53−/−^ cells, AdoMet treatment affected cell cycle, causing a marked arrest of cell cycle at the S phase, where this effect was accompanied by a strong increase of cyclin E. Furthermore, a downregulation of cyclin D expression was also observed in these cells. It is known that the overexpression of cyclin D results in drug resistance in different cancers [46]. We have previously demonstrated a role of cyclin D in uL3-mediated drug resistance showing that its increased expression may contribute to cellular chemoresistance in uL3∆HCT 116^p53−/−^ cells [10]. We believe that the downregulation of cyclin D upon AdoMet treatment observed only in uL3∆HCT 116^p53−/−^ cells is responsible for the resensitizing of the cells to anticancer treatments. This is in accordance with the view that targeting and inhibiting cyclin D is an effective strategy to overcome drug resistance.

In addition to its role in cell cycle regulation, cyclin D is also involved in the regulation of apoptosis. Impairing apoptosis is a critical point in the development of drug resistance [47]. Our data demonstrated that the exposure of uL3∆HCT 116^p53−/−^ cells to AdoMet induced apoptosis and this was in accordance with the ability of AdoMet to downregulate cyclin D1 level.

Caspases are aspartate-specific cysteine proteases that play crucial roles in apoptosis [48]. Activation of caspases results in the cleavage and the inactivation of key cellular proteins including the DNA repair enzyme poly(ADP ribose) polymerase 1 (PARP-1). Our results showed that AdoMet caused a downregulation of PARP-1 associated to the cleavage of pro-caspase 3, which was specifically mediated by caspase 9. In addition, apoptosis was also evaluated by measuring the ratio between specific anti-apoptotic and pro-apoptotic proteins. We found that AdoMet treatment caused a strong reduction in anti-apoptotic Bcl-2 levels, favoring the expression of pro-apoptotic Bad. In addition to its canonical anti-apoptotic activity, Bcl-2 has also been implicated in the regulation of ROS levels in the mitochondrion [49]. Several studies suggest a reciprocal relationship between ROS and Bcl-2 levels within cells; increases in ROS correlate with decreases in Bcl-2 levels and vice versa [49]. In this study, we reported that AdoMet treatment is associated with an increase in ROS production and a downregulation of Bcl-2 expression in both cell lines.

It has been largely demonstrated that defects of autophagy are associated with an increase of ROS, which, in turn, can activate the intrinsic apoptotic pathway [50].

Autophagy has been defined as a ‘double-edged sword’ in cancer progression; it may act as a tumor suppressor by clearing damaged organelle, preventing oxidative stress and genomic instability, particularly during malignant transformation and carcinogenesis. However, in established tumors, it may act as a pro-survival pathway during stress conditions like nutrient deprivation, hypoxia, and cancer therapeutic treatment. Moreover, the inhibition of autophagy has been shown to prevent resistance of cancer cells to chemotherapy [36]. Recently, we have shown that resistance to drugs caused by uL3 depletion in p53-deleted colon cancer cells was due to autophagy induction [11].

An involvement of AdoMet in autophagy regulation has already been demonstrated in breast cancer cells. In MCF-7 cells, AdoMet showed a synergistic effect with chloroquine (CQ) in the inhibition of autophagy through the targeting of the AKT/mTOR pathway [29]. Our results demonstrated that AdoMet treatment inhibited the autophagy flux in uL3∆HCT 116^p53−/−^ cells, characterized by a high basal protective autophagy. Consistently, a reduction in LC3BII/I ratio and Atg7 levels, and an increase in p62 amount were found. Since it has been demonstrated that p62 overexpression leads to caspase-8-dependent apoptosis [51], our results suggest that inhibition of autophagy caused by AdoMet leads to an increase of apoptosis, promoting tumor cell death.

## 4. Materials and Methods

### 4.1. Materials

PI, MTT, 2′,7′-Dichlorofluorescein (DCF), CQ, and 5-FU were purchased from Sigma-Aldrich (St. Louis, MO, USA). Bovine serum albumin (BSA), fetal bovine serum (FBS), Dulbecco’s modified Eagle’s medium (DMEM), phosphate-buffered saline (PBS), and trypsin-EDTA were obtained from Gibco (Grand Island, NY, USA). Tissue culture dishes were purchased from Corning (Corning, NY, USA). AdoMet was provided from New England Biolabs, prepared in a solution of 5 mM H_2_SO_4_ and 10% ethanol, filtered, and stored at 4 °C until use., LTR and the Annexin V-fluorescein isothiocyanate (V-FITC) Apoptosis Detection Kit were purchased from eBioscience (San Diego, CA, USA). Monoclonal antibodies (mAb) to cyclin D1, cyclin E1, cyclin A2, pro-caspase 9, pro-caspase 8, poly(ADP ribose) polymerase 1 (PARP-1), Bcl-2, Atg7, β-actin, α-tubulin and polyclonal antibodies (polyAb) to cyclin B1, pro-caspase 3, Bad, LC3BI/II, and p62 were purchased from Cell Signaling Technology (Danvers, MA, USA). Horseradish peroxidase (HRP)-conjugated goat anti-mouse (GxMu-003-DHRPX) and HRP-conjugated goat anti-rabbit (GtxRb-003-DHRPX) secondary antibodies were obtained from ImmunoReagents. Inc., (Raleigh, NC, USA). All buffers and solutions were prepared with ultrahigh quality water. All reagents were of the purest commercial grade. 

### 4.2. Cell Cultures and Drug Treatments

HCT 116^p53−/−^ cells and uL3ΔHCT 116^p53−/−^, a cell line derived from HCT 116^p53−/−^ cell and stably silenced for uL3 [17], were cultured at 37 °C in a 5% CO_2_ humidified atmosphere and grown in Dulbecco’s modified Eagle’s medium (DMEM) supplemented with 10% fetal bovine serum (FBS), 2 mM L-glutamine, and penicillin-streptomycin 50 U/mL. Typically, subconfluent cells were seeded into tissue culture plates and treated with 500 µM AdoMet for 24, 48, and 72 h.

### 4.3. Cell Viability Assays

HCT 116^p53−/−^ cells and uL3ΔHCT 116^p53−/−^cells were plated in serum-containing media in 96-well plates at the proper density. After 24 h incubation, the cells were treated with increasing concentrations of AdoMet (from 72 to 1000 μM) for 24, 48, and 72 h. Cell viability was assessed by adding MTT as previously reported [52]. The absorbance values of the solution in each well were detected at 570 nm using a Bio-Rad IMark microplate reader (Bio-Rad Laboratories, Milan, Italy). All MTT experiments were performed in quadruplicate. Cell viability was expressed as the percentage of absorbance values in treated samples with respect to that of the control (100%). For the treatments, AdoMet plus 5-FU, uL3ΔHCT 116^p53−/−^ cells were seeded at proper density in 96-multiwell plates. After 24 h incubation, the cells were treated with different concentrations of AdoMet (from 125 to 1000 µM) and 5-FU (12.5 to 100 µM), alone or in combination, for 72 h and cell viability was detected by the MTT assay.

### 4.4. Cell Cycle Analysis

HCT 116^p53−/−^ cells and uL3ΔHCT 116^p53−/−^ cells were seeded into 35 mm tissue culture plates at a confluency about 50–60%. Then, cells were starved overnight and treated with 500 µM AdoMet for 72 h. After treatment, the cells were harvested and centrifuged at 400× *g* for 5 min, washed once with cold PBS and stained in a PI solution as previously reported [53]. Flow cytometric analysis was performed using a BD Accuri™ C6 flow cytometer (BD Biosciences). To evaluate cell cycle progression, PI fluorescence was collected as FL3-A (linear scale) using ModFIT software (Verity Software House). For each sample, at least 20,000 events were analyzed in at least three different experiments, giving a standard deviation (SD) < 5%.

### 4.5. Cell Death Assay

HCT 116^p53−/−^ and uL3ΔHCT 116^p53−/−^ cells were seeded in 6-well plate, starved overnight, and treated with 500 µM AdoMet for 72 h. The cells were harvested by trypsinization and washed twice with PBS. Annexin VFITC was used in conjunction with the vital dye PI to distinguish apoptotic (Annexin VFITC positive, PI positive) from necrotic (Annexin VFITC negative, PI positive) cells as previously reported [54]. Briefly, cells were re-suspended in 200 μL of Binding Buffer 1X and incubated with 5 μL Annexin V-FITC and 10 μL PI (20 μg/mL) for 30 min at room temperature, as recommended by the manufacturers. The detection of viable cells, early apoptotic, late apoptotic, and necrotic cells was performed by BD Accuri™ C6 flow cytometer (BD Biosciences). For each sample, 20,000 events were recorded. Analysis was carried out by triplicate determination on at least three separate experiments. 

### 4.6. Determination of ROS by the 2′,7′-dichlorofluorescein Diacetate (DCFH-DA) Assay

Colon cancer cells were seeded in 6-well plates at the proper density, and after 24 h of incubation, the cells were treated with AdoMet 500 µM for 72 h. The positive control was represented by the cells treated with menadione at the final concentration of 100 μM for 1 h at 37 °C. After treatment, cells were stained with 10 μM DCF-DA for 30 min at 37 °C in the dark [55]. DCFH-DA can freely pass the cell membrane where it is trapped after being hydrolyzed by intracellular esterases in a non-fluorescent compound (DCFH). Then, DCFH can be oxidized to the fluorescent DCF by reacting with intracellular ROS. Following incubation, cells were washed twice with PBS, trypsinized, resuspended in PBS, and immediately analyzed with a BD Accuri™ C6 flow cytometer (BD Biosciences). For each sample, 20,000 events were recorded. Analysis was carried out by triplicate determination on at least three separate experiments. 

### 4.7. LysoTracker-Red Staining

HCT 116^p53−/−^ and uL3ΔHCT 116^p53−/−^ cells were seeded in a 6-well plate and treated with 500 μM AdoMet for 48 h, using CQ as the positive control. LTR was added to each well for 20 min at 37 °C at a final concentration of 0.1 μM in medium. The cells were then washed with PBS and observed by fluorescence microscopy. The fluorescence intensity of the cells was then analyzed by flow cytometry. Briefly, the cells were detached by incubation with EDTA-trypsin, washed twice with PBS, and collected by centrifugation. For the quantitative evaluation of LTR, FlowJo software was used to calculate the median fluorescence intensities (MFI) by the formula (MFI-treated/MFI-control), where MFI-treated is the fluorescence intensity of cells treated with the various compounds and MFI-control is the fluorescence intensity of the untreated cells. For each sample, 20,000 events were acquired. Analysis was carried out by triplicate determination on at least three separate experiments.

### 4.8. Western Blot Analysis

After the treatment time, cells were harvested and protein extracts were prepared as previously described [56]. A western blot analysis was performed as previously reported [57]. All primary antibodies were used at a dilution of 1:1000; all secondary antibodies were used at a dilution of 1:5000. Blots were then developed using enhanced chemiluminescence detection reagents ECL (Cyanagen, Bologna, Italy) and exposed to X-ray film. All films were scanned by using Image J software (National Institutes of Health, Bethesda, MD, USA). 

### 4.9. Statistical Analysis

Experiments were performed at least three times with replicate samples. Data are expressed as mean ± standard deviation (SD). *p*-values were determined using unpaired two-tailed Student’s *t*-test. * *p* < 0.05 was considered significant, ** *p* < 0.01 was considered highly significant.

## 5. Conclusions

**Figure 7:**
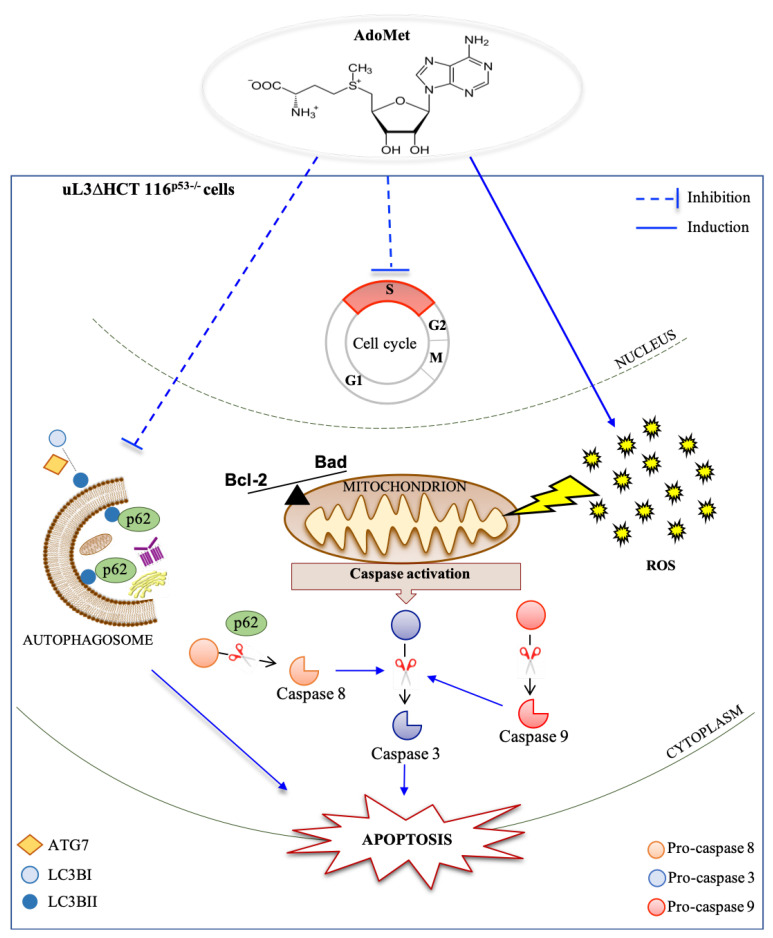
Schematic representation of the proposed model of AdoMet effects in uL3∆HCT 116^p53−/−^ colon cancer cells. AdoMet treatment affected cell proliferation causing cell cycle arrest in the S phase. The inhibition of the autophagic process and induction of ROS upon exposure to AdoMet led to activation of the caspase cascade, triggering the apoptotic pathway.

All these data led us to propose the model shown in Figure 7 in which AdoMet, by targeting multiple pathways, overcomes the drug resistance of p53-deleted colon cancer cells expressing low levels of uL3. In particular, AdoMet blocks cell cycle at the S phase, inhibits autophagy, augments ROS species, and finally triggers the activation of the apoptotic pathway.

Overall, our findings highlighted AdoMet, a naturally-occurring multifunctional sulfonium compound, as a potential candidate for novel therapeutic approaches to overcome the drug resistance of colon cancer depending on p53 and uL3 status.

## Supplementary Materials

The following are available online at https://www.mdpi.com/1422-0067/22/1/103/s1.

## Abbreviations

AdoMet	S-adenosyl-l-methionine
MDR	multidrug resistance
5-FU	5-fluorouracil
ROS	reactive oxygen species
DCF-DA	2′,7′-dichlorofluorescein diacetate
MFI	median intensity fluorescence
LTR	LysoTracker
CTR	untreated cells
CQ	chloroquine
FBS	fetal bovine serum
DMEM	Dulbecco’s modified Eagle’s medium
MTT	3-(4, 5-dimethylthiazol-2-yl)-2, 5-diphenyltetrazolium bromide
PI	Propidium iodide
PBS	phosphate-buffered saline
Annexin V-FITC	Annexin V-fluorescein isothiocyanate
