# Supplementary material for: S-Adenosyl-l-Methionine Overcomes uL3-Mediated Drug Resistance in p53 Deleted Colon Cancer Cells
Source: Int J Mol Sci. 2020 Dec 24;22(1):103. doi: 10.3390/ijms22010103 (PMC7795960; doi:10.3390/ijms22010103)
Supplement: Supplementary file 1 [file ijms-22-00103-s001.pdf]

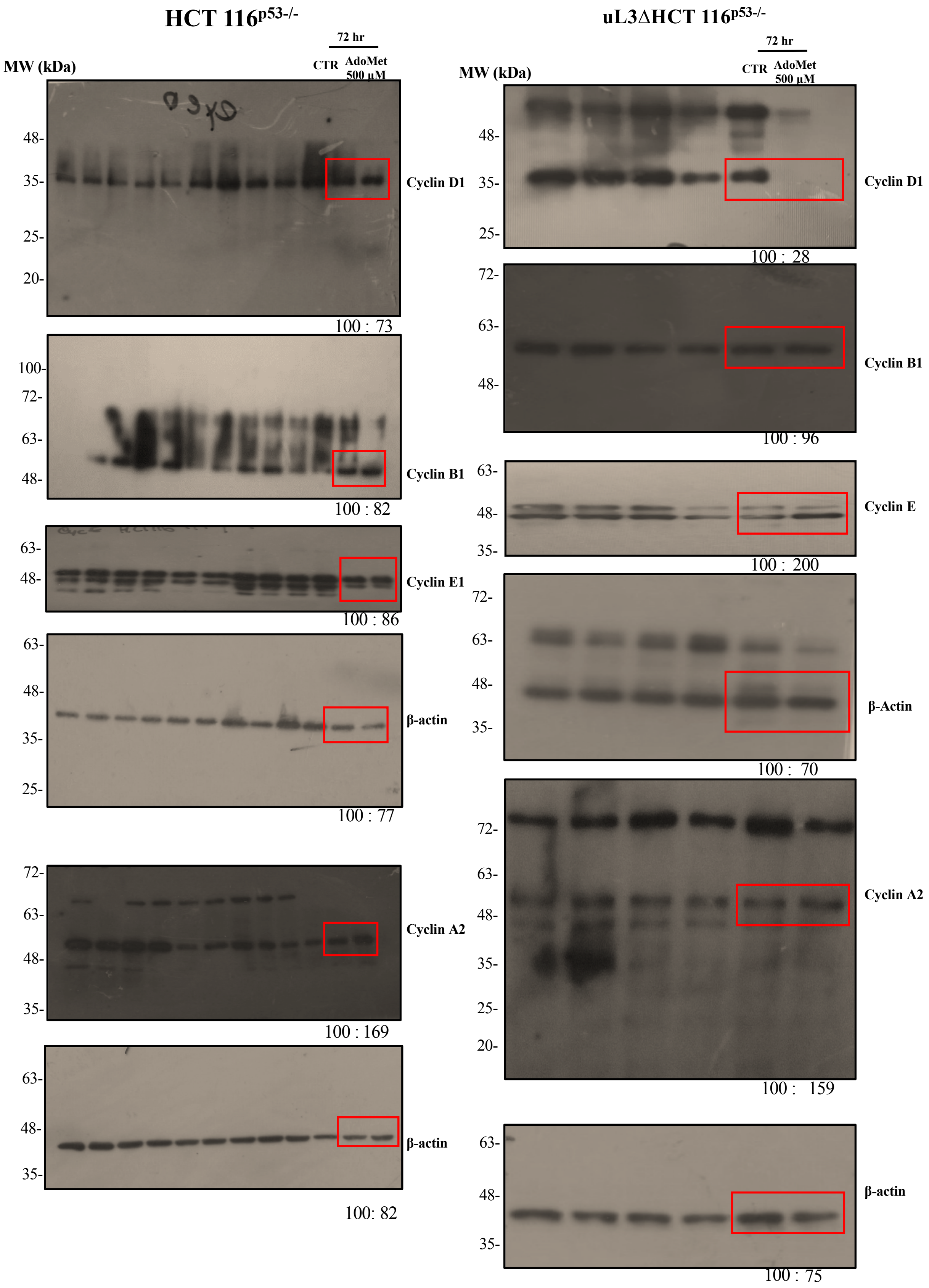

Figure S1. Effect of AdoMet on cell cycle regulatory proteins in HCT 116$^{p53-/-}$ and uL3ΔHCT 116$^{p53-/-}$ colon cancer cells. The cropped blots are used in the main figure (Figure 3).

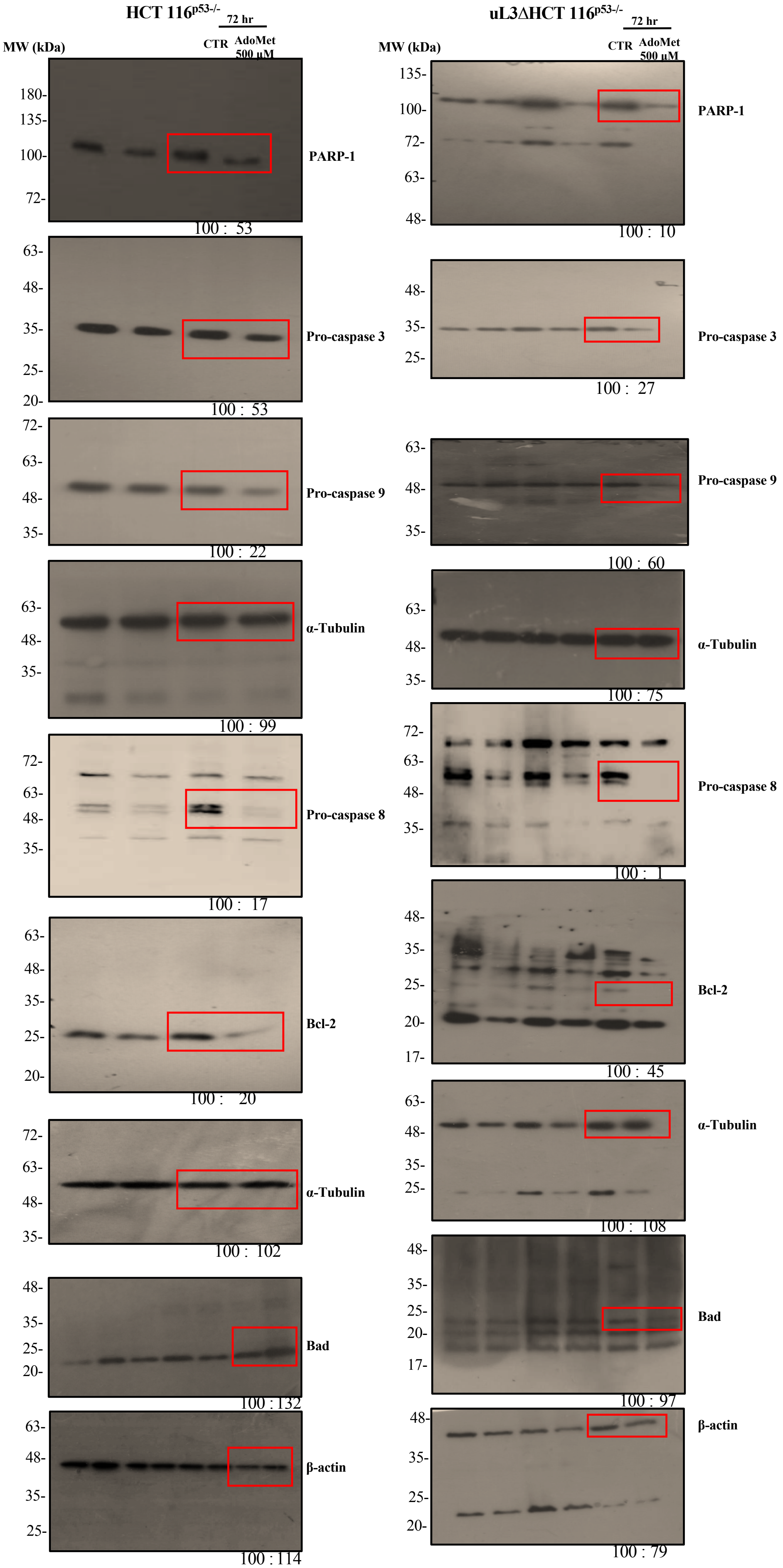

Figure S2. Effect of AdoMet on apoptosis in HCT 116$^{p53-/-}$ and uL3ΔHCT 116$^{p53-/-}$ colon cancer cells. The cropped blots are used in the main figure (Figure 4).

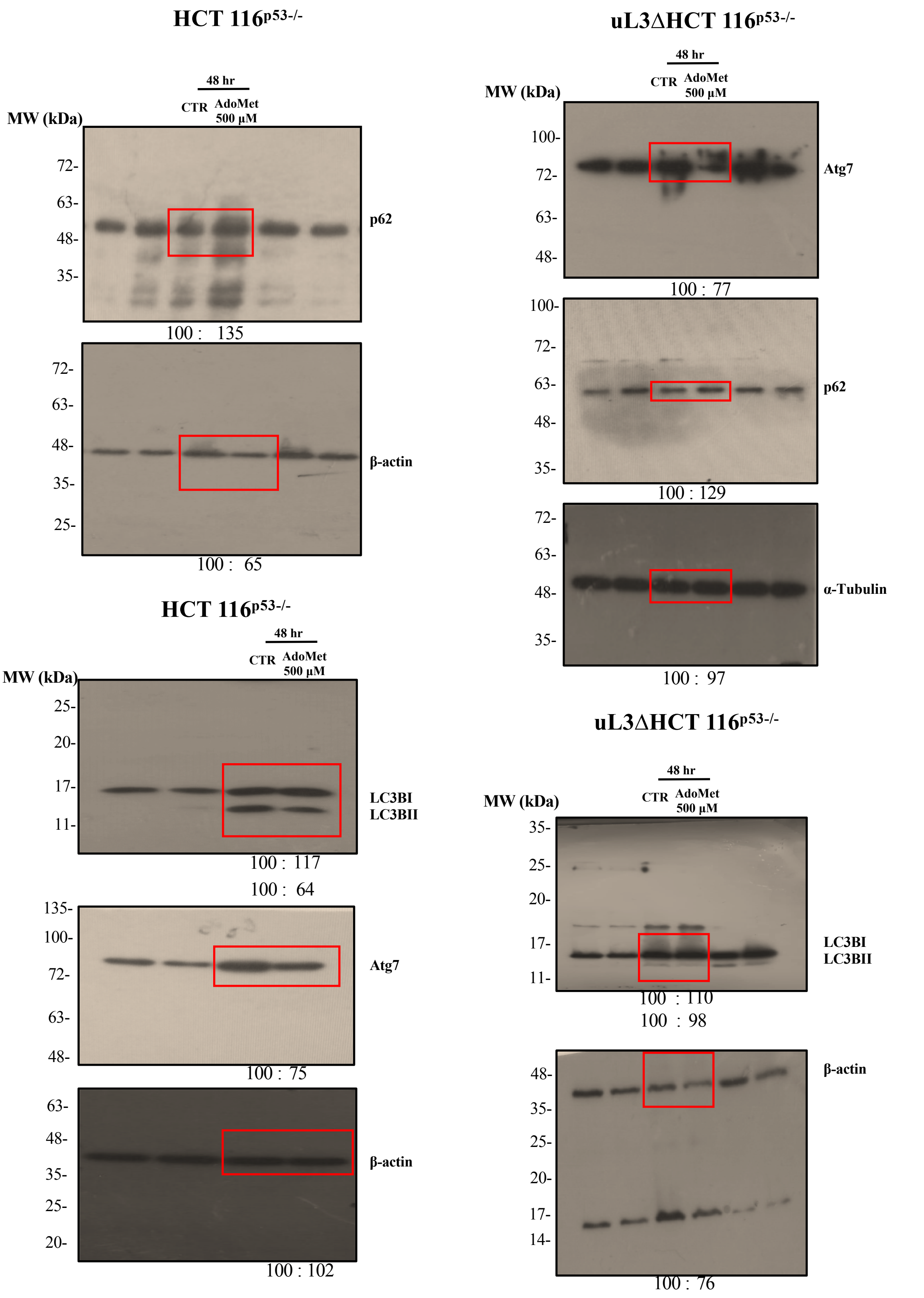

Figure S3. Effect of AdoMet on the autophagy related markers in HCT $116^{p53-/-}$ and uL3ΔHCT $116^{p53-/-}$ colon cancer cells. The cropped blots are used in the main figure (Figure 6).
